# Dataset of the construction and characterization of stable biological nanoparticles

**DOI:** 10.1016/j.dib.2020.106536

**Published:** 2020-11-14

**Authors:** Romina A. Gisonno, M. Alejandra Tricerri, Marina C. Gonzalez, Horacio A. Garda, Nahuel A. Ramella, Ivo Díaz Ludovico

**Affiliations:** a Instituto de Investigaciones Bioquímicas de La Plata (INIBIOLP), Argentina; b Facultad de Ciencias Médicas, Universidad Nacional de La Plata, Calle 60 y 120, La Plata CP 1900, Argentina

**Keywords:** Apolipoprotein A-I, BS^3^ crosslinker, Lipid-binding, Gradient gel electrophoresis, Nanoparticles

## Abstract

This article shows the dataset of clearance assays and the reconstitution of stable biological nano-complexes using both detergent-assisted and spontaneous solubilization of phospholipids by the recombinant purified apolipoprotein A-I (apoA-I). Protein was intra-chain crosslinked in order to introduce steric constrains. Then, native and crosslinked protein function was evaluated by a data collection of dimiristoyl phosphatidyl choline (DMPC) micellization curves. Additionally, resulting particles from spontaneous or detergent-assisted lipid solubilization were characterized by transmission electron microscopy (TEM), size exclusion chromatography (SEC), and native polyacrylamide gel electrophoresis (PAGE). Here we set up an experimental design that may help study protein structure based on its function, since interaction with biological membranes and lipids is an intrinsic activity attributed to many proteins in circulation. In addition, by *t*-test analysis of collected-data, we examined the formation of lipoprotein particles by native and intra-chain crosslinked proteins under different conditions like temperature and time incubation. Thus, data shown here strengthen the usefulness of an easy, rapid, accessible and inexpensive approach to test protein flexibility related to its function.

## Specifications Table

**Table:** 

Subject	Biochemistry and Biophysics
Specific subject area	Biophysics (lipid/protein assay) Protein structure and lipid interaction
Type of data	Image Graph Figure
How data were acquired	The lipid-protein interaction studies were carried out through native polyacrylamide gradient gel electrophoresis (PAGE). Gels were run in a Mini-PROTEAN® (BIO-RAD Tetra Vertical Electrophoresis Cell for Mini Gels, 4-gel #1658004). Intensity associated with the bands was quantified with ImageJ software version 1.51 j8. Micellization assays were monitored alternatively in a Helios Beta: Single beam, quartz coated (Thermo) or in a UV–visible Agilent Cary 8454 (Agilent Technologies) spectrophotometers kinetics curves were obtained using SigmaPlot 12.0 (Systat Software, Inc.) Statistical analysis was performed by the GraphPad Prism version 8.0.0 for Windows (GraphPad Software, San Diego, California USA, www.graphpad.com). The relative size of the particles was estimated by both native PAGE and size exclusion chromatography (SEC). Samples were resolved by elution through a 6 HR column (Pharmacia) using a Merck-Hitachi L6200 Intelligent pump and detected at 280 nm by a UV–VIS detector (Merck-Hitachi L4200). Particle morphology was characterized by transmission electron microscopy (TEM) on a JEOL-1200 EX microscope and observed under negative staining.
Data format	Raw Analyzed Filtered
Parameters for data collection	Data were collected by three or five independent experiments using recombinant proteins and commercial lipids.
Description of data collection	Each dimyristoyl phosphatidyl choline (DMPC) solubilization kinetics was normalized to the absorbance at minute 0 at λ_325_ _nm_. Quantification of the relative amount of the reconstituted lipoproteins was performed by scanning all the bands within each lane and measuring the intensity of each band with relation to the 100% of the total intensity.
Data source location	Institution: Instituto de Investigaciones Bioquímicas de La Plata (INIBIOLP). CCT CONICET La Plata City: La Plata, Buenos Aires Country: Argentina
Data accessibility	With the article Direct URL to data: Díaz Ludovico, Ivo; Ramella, Nahuel A. (2020), “Dataset of the construction and characterization of stable biological nanoparticles”, Mendeley Data, V2, http://dx.doi.org/10.17632/rgk9n9wt3d.1

## Value of the Data

• Data show a robust experimental approach to construct stable nanoparticles which may help test protein flexibility and might act as platforms to be used as carriers of drugs or biological compounds.
• These data may benefit the extended field of either basic or applied biochemistry research, as it may give information on the protein structure-function relationship. Its simplicity and low cost will surely make it possible an extensive use.
  This design may be combined with mass spectrometry, and fluorescence techniques to characterize the spatial arrangement of proteins within the platforms and the domains that may interact with membranes.
• Data show a well-defined methodological design to evaluate the interaction of flexible proteins with different lipid microenvironments and the importance of a structural constraint as induced here by intra-chain crosslinking.

## 1 Data Description

**Fig. 1:**
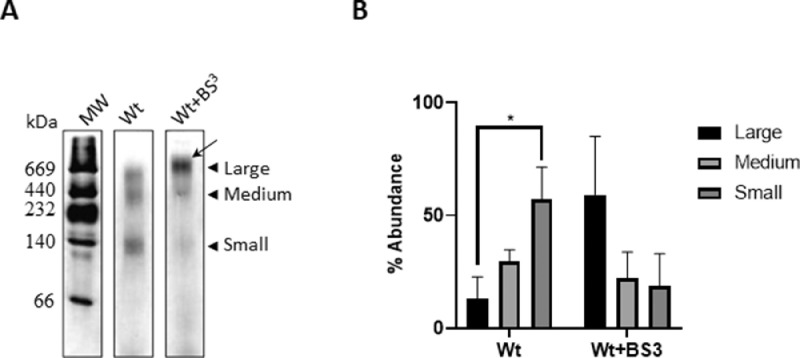
Characterization of sodium cholate-mediated reconstituted particles.

The efficiency of protein solubilization as lipid complexes and the effect of crosslinking in this behavior were initially evaluated by using detergent-mediated interaction [1]. In this regard, human apoA-I reconstituted into discoidal complexes (rHDL) was previously used in clinical trials to vehiculize cholesterol out of the atherosclerotic plaques [2] or as drug carriers, since the structural arrangement resembles that of the particles isolated *in vivo* [3]. These particles may be attained by using sodium cholate, an amphipathic molecule that was shown to promote the formation of bilayer-like complexes due to its flat-shaped structure [1]. ApoA-I with the native sequence (Wt), either unmodified (native) or crosslinked (Wt+BS^3^), was incubated with DMPC multilamellar vesicles (MLV) at a 40:60:1 lipid:sodium cholate:protein molar ratio and the product of the rearrangement of the reconstituted particles tested and characterized. Fig. 1 shows that under our conditions Wt rearranged mainly as three discretely-sized rHDL of approximate 140, 440 and 670 kDa molecular weight (Fig. 1A). The relative quantification of the intensity associated with the bands after gel imaging was performed in the ImageJ software. Then, intensities were normalized to the sum of intensities for each lane and shown in Fig. 1B as arbitrary intensity units (as shown in the online repository, along with the original figures). These data confirmed a higher yield of the smallest population (Fig. 1B). Instead, intra-chain crosslinked Wt (Wt+BS^3^) yielded a particle population, mainly represented by the largest complexes (indicated in Fig. 1A by the black stealth arrow).

Wt and Wt+BS^3^ were incubated with DMPC MLV at a molar ratio 40:1 in the presence of sodium cholate as indicated in Methods. A) Home-made 4–30% non-denaturing, gradient gel electrophoresis (PAGE) developed with silver staining. The apparent molecular weight was evaluated by comparison with high molecular weight commercial standards (labeled to the left). B) The relative amount of each population was estimated by quantifying the intensity of the different bands in each lane. Bars represent media ± standard deviation of triplicates of three independent measurements as evaluated by t-test. Symbol * indicates significant differences at *p* ≤0.05. Densitometry measurements determined by ImageJ, are available in the online repository, as raw and normalized values (https://data.mendeley.com/datasets/rgk9n9wt3d/1/files/1661c901-454d-401c-ba9b-008de37e1c32).

**Fig. 2:**
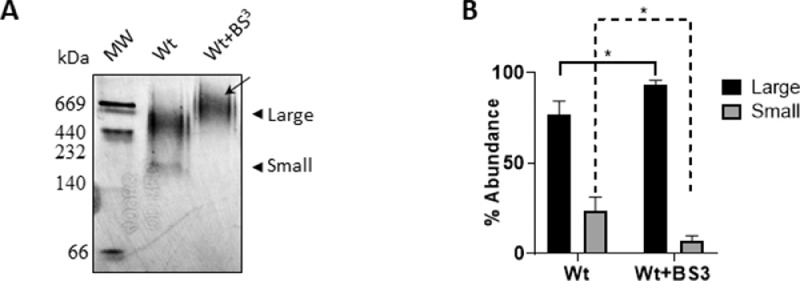
Characterization of the spontaneous interaction of Wt with DMPC.

In a different design, interactions of Wt with lipids may occur at the lipid transition temperature (Tm), where the solubilization of phospholipids by apoA-I was previously shown to be maximized [4]. The convenient Tm of the DMPC (24 °C) is well suited to perform this test without requiring sophisticated lab heaters and avoiding proteins to be incubated under drastic conditions. Thus, we set to characterize the effect of intra-molecular crosslinking by incubating Wt and Wt+BS^3^ with DMPC MLV for 3 h at 24 °C. First, we analyzed the product of this interaction. As Fig. 2 A shows, under these conditions large discretely-sized rHDL were obtained from Wt, with a low amount (around 20% as it is observed in Fig. 2B) of smaller complexes by using the same methodology for data collection as described above and the raw data from gel densitometry measurements its available in the online repository (https://data.mendeley.com/datasets/rgk9n9wt3d/1/files/a0b7b063-169c-42b9-8fb9-ea6d906a8c38). Instead, and similarly to data shown for sodium cholate-mediated rHDL, Wt+BS^3^ yielded mostly one large population (indicated as in Fig. 1 by the black stealth arrow).

Multilamellar DMPC vesicles were incubated with Wt (0.05 mg/mL) or Wt+BS^3^ at a molar ratio 145:1 DMPC: protein at 24 °C in phosphate saline buffer (PBS) pH 7.4 for 3 h (A) The relative size of the incubation product was estimated by 4–30% non-denaturing gradient gel electrophoresis (PAGE) developed with silver staining as explained above. From left to right, commercial Mw marker, Wt and Wt+BS^3^; B) The relative amount of each population was estimated by quantifying the intensity of the different bands in each lane. Bars represent media ± standard deviation of triplicates of three independent measurements as evaluated by *t*-test. Symbol * indicates significant differences at *p*≤0.05 between Wt or Wt+BS^3^ large particles (solid line) or small complexes (dashed line).

**Fig. 3:**
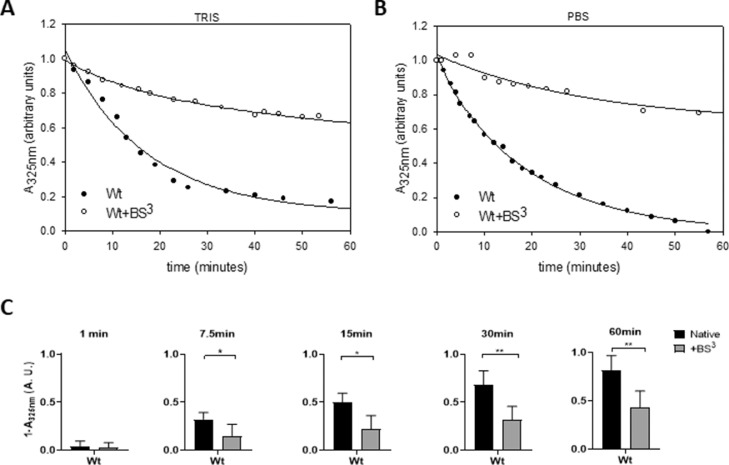
Analysis of the kinetics of DMPC clearance mediated by Wt.

As the simplicity of this procedure makes it highly accessible to a vast biochemical research field, we further characterized its properties. To this regard, we evaluated the importance of the regular buffer compositions used in a lab routine to perform this assay (either Tris or PBS) on the efficiency of protein arrangement. As it is well known, the efficiency of the interaction may be followed by the decrease of the lipid turbidity as large MLV rearrange into small nano discs with lower light dispersion. Each point was registered as raw arbitrary absorbance unit in a spectrophotometer at 325 nm. Points were then normalized to sample absorbance at time 0 (or what it is equivalent immediately after addition of MLV) using only DMPC MLV, in the corresponding buffer as turbidity control. Each dataset of point distributions was fitted using exponential decay function as we detailed in “DMPC clearance assay” section. From the registry of the absorbance during the incubation time, (and as it was previously reported [5]), Wt clarified MLV with fast kinetics (dark symbols in Fig. 3 A). Instead, crosslinked protein exhibited a lower kinetics to clearance, (white symbols). No difference between curves was observed with Tris or PBS buffer. To better characterize the comparative clearance progression, Wt and Wt+BS^3^ fitted function was analyzed statistically at different times. Even though the lower kinetics of the Wt+BS^3^ was observed from the beginning of the reaction, it became significant after 7.5-min incubation under these conditions (Fig. 3C). Data from clearance assays are available in the online repository as arbitrary absorbance values, point by point, as normalized values (https://data.mendeley.com/datasets/rgk9n9wt3d/1/files/de4bea35-3b38-4fe4-9254-ceeeac96c9f7).

DMPC multilamellar vesicles were incubated with Wt (0.05 mg/mL) or crosslinked Wt (Wt-BS^3^) at a molar ratio 145:1 DMPC: protein at 24 °C, either in Tris 20 mM (A) or PBS (B) buffers, both at pH 7.4 for 1 h; time dependence of the absorbance at 325 nm was monitored for 1 h. C) Difference in the micellization efficiency between Wt (black bars) or Wt-BS^3^ (grey bars) was estimated from the change in the absorbance at each incubation time. Bars represent media ± standard deviation of triplicates of three independent measurements as evaluated by *t*-test. Symbols * and ** indicate significant differences at *p* ≤0.05 and *p* ≤ 0.01, respectively.

**Fig. 4:**
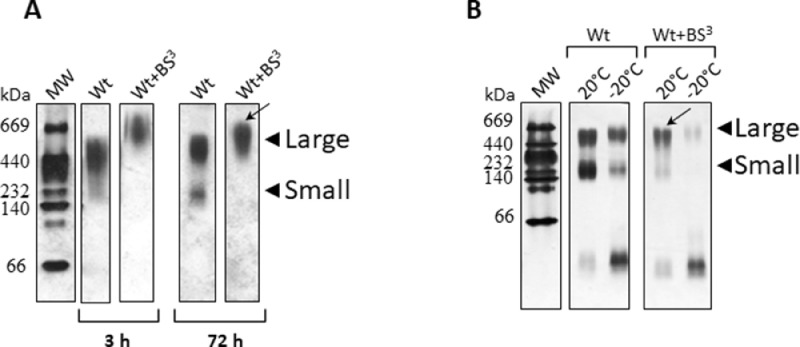
Stability of Wt complexes as a function of incubation time and temperature.

Next, we set to characterize the effect of incubation time on the stability of the rearranged nanoparticles. When we compared particle stability at 24 °C at 72 h versus 3 h [5], longer times resulted in a small arrangement of Wt, yielding some degree of smaller particles (Fig. 4A).Instead, Wt+BS^3^ remained mainly as a unique discrete, larger population (indicated by a black arrow).

Afterward, we analyzed the stability of the rearranged rHDL by keeping samples at different temperatures. This control is worth to be done, as lipid-protein interactions are strongly dependent on temperature. Twenty Celsius degrees was chosen as it is a normal lab room temperature. Instead, freezing at −20 °C is important to be evaluated, as storage at low temperatures may be a common procedure to preserve samples from deterioration. Particles obtained after 72 h at 24 °C (Fig. 4)  were incubated for 7 days at 20 °C or −20 °C, alternatively. A higher yield of the smaller particles and some amount of lipid-free protein were observed at 20 °C incubation for Wt. A lower effect was observed for Wt+BS^3^ on lipoprotein distribution (Fig. 4B, labeled as above by the black stealth arrow). Instead, for the case of lipid-protein complexes incubated under freezing conditions, major amount of lipid-free proteins was observed for both Wt and Wt+BS^3^ visualized in Fig. 4B as a single band with a molecular weight minor to 66 kDa.

DMPC MLV were incubated with Wt (0.05 mg/mL) or Wt+BS^3^ at a molar ratio 145:1 DMPC: protein at A) 24 °C for 3 h or 72 h; B) Particles obtained after 72 h at 24 °C (as shown in A) were incubated for 7 days either at 20 °C or −20 °C. The relative size of the incubation product was estimated by 4–30% non-denaturing gradient gel electrophoresis (PAGE) developed with silver staining as explained above.

**Fig. 5:**
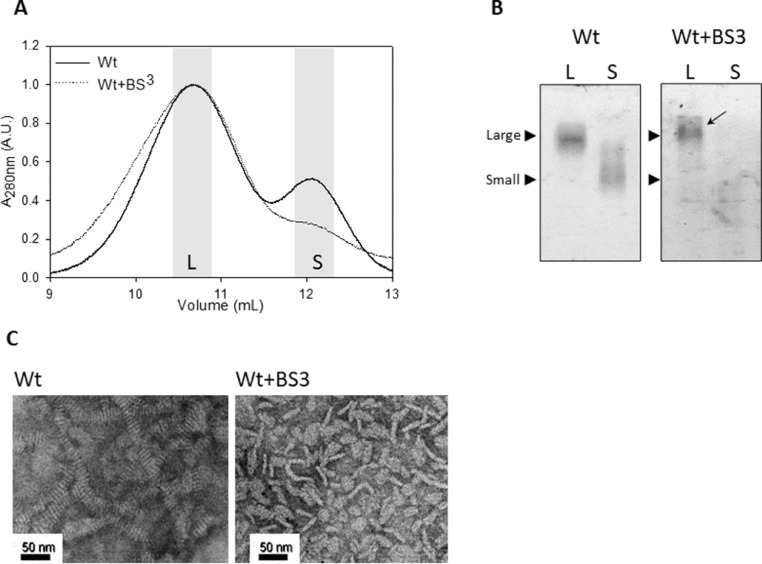
Characterization of apoA-I variants rearrangement.

Finally, the different complexes obtained by incubation for 72 h at 24 °C were isolated by size exclusion chromatography (Fig. 5A), and reanalyzed afterwards by native PAGE (Fig. 5B). At the online repository we submitted the raw and normalized 280 nm absorbance chromatograms as a point by point dataset (https://data.mendeley.com/datasets/rgk9n9wt3d/1/files/2a791058-38eb-4db2-af91-86ca17b5226a). Most of the protein eluted as large complexes, especially in the case of Wt+BS^3^, which remained stable under the elution and concentration steps, as shown in Fig. 3B. The analysis of complex morphology by TEM indicated that Wt formed the well-known disc-shaped complexes [1]. Crosslinked Wt also formed discoidal particles but with some elongated conformations (Fig. 5C). The raw TEM imaging are available in the online repository as a PDF file (https://data.mendeley.com/datasets/rgk9n9wt3d/1/files/9d938b70-c512-46ce-955b-ee11b4c3ae8a)

Wt and Wt+BS^3^ (at a protein concentration of 0.05 mg/mL) were incubated with DMPC for 72 h at 24 °C at a lipid-to-protein molar ratio of 145:1 and the product of the micellization analyzed. A) Size exclusion chromatography was performed through a Superose 6 HR column (Pharmacia), equilibrated with 50 mM Tris buffer pH 7.4 at a flow of 0.5 mL/min. Collected fractions corresponding to the main peaks (shaded in grey) were pooled, concentrated and analyzed by a 4–30% non-denaturing, home-made gradient gel electrophoresis developed with silver staining B). Homogenous large sized Wt-BS^3^complexes are labeled by a black stealth arrow; C) Morphology of the reconstituted HDL from Wt and Wt-BS^3^ was analyzed by TEM under negative staining prior to FPLC isolation. Black bars in C) indicate the magnification scale used for the observations.

## 2 Experimental Design, Materials and Methods

### 2.1 Materials

Guanidinehydrochloride (GndHCl), cholesterol (Chol), sodium cholate, ethylenediaminetetraacetic acid (EDTA) and sodium chloride (NaCl) were purchased from Sigma Chemical Co. (St Louis, MO); dimyristoyl phosphatidylcholine (DMPC) was purchased from Avanti Polar Lipids. Alabaster, AL. His-purifying resin was from Novagen (Darmstadt, Germany). Bis-(sulfosuccinimidyl) suberate (BS^3^) and isopropyl-β-D-thiogalactoside (IPTG) were purchased from Thermo Scientific (Waltham, MA). All other reagents were of the highest analytical grade available.

### 2.1 Protein variants purification

ApoA-I was expressed from a bacterial system transformed with a cDNA containing the native human sequence further modified to introduce an acid-labile Asp-Pro peptide bond between amino acids 2 and 3 [6]. This construct was inserted into a pET-30 plasmid (Novagen, Madison, WI), transformed into BL21 (DE) *E coli* cells (Novagen, Madison, WI) grown in Luria-Bertani (LB) medium in presence of kanamycin at 50 µ/mL, then expressed by induction with IPTG 0.4 mM. Bacteria were harvested by centrifugation and lysed by sonication in Tris-buffered saline with 3M guanidine hydrochloride and purified using immobilized metal affinity chromatography (IMAC) by elution through Ni-chelating columns (Novagen, Madison, WI).  Fractions containing His-tagged-apoA-I were pooled and dialyzed. Then, N-terminal His-Tag extension was removed by incubation of isolated apoA-I in 50% formic acid (v/v) for 5 h at 55 °C. Proteins were then dialyzed at 4 °C until formic acid removal, and then at least 2 buffer changes against Tris-buffer saline at 4 °C (each at 1:1,000 v/v protein:buffer). Finally, the protein was separated from the tag, eluting it through the Ni-chelating column [7]. This resulted in a high yield of protein with a purity of at least 95% (determined by SDS-PAGE). Samples were stored at −80 °C with 3M guanidine hydrochloride and extensively dialyzed (3 buffer changes each at 1:1000 v/v protein:buffer) of the corresponding buffer prior to use.

### 2.2 Protein Crosslinking

Proteins were crosslinked at 0.05 mg/mL (monomeric state) in PBS pH 7.9 for 3 h without agitation at room temperature. Fresh BS^3^ was added (within 1 min after solubilization in PBS to avoid hydrolysis of free crosslinker) at 30:1 BS^3^:protein molar ratio [8]. Reactions were quenched for 15 min after the addition of Tris buffer to a 50 mM final concentration. The concentration of crosslinked proteins was calculated by the BCA Protein Assay Kit (Thermo Scientific (Waltham, MA)). The presence of the monomeric conformation after the treatments was confirmed by polyacrylamide gradient electrophoresis, either under native or denaturing conditions. Oligomers were not observed at these conditions. Proteins were dialyzed against PBS or TRIS buffer at pH7.4, alternatively and stored at −20 °C.

### 2.3 DMPC multilamellar vesicles construction

DMPC (5 mg from a stock solution in chloroform) was used to form a film in a round-bottom tube, dried by blowing a N_2_ atmosphere and exhaustively exposed to vacuum in a lyophilizer (Virtis) to evaporate the solvent. Then, PBS or Tris buffers at pH 7.4 were added to a final DMPC concentration of 5 mg/mL. MLV were attained by extensive vortexing at room temperature for 5 min, then heating at 37 °C in three cycles of 30 s each [9].

### 2.4 DMPC clearance assay

DMPC MLV were added to the 0.05 mg/mL protein samples (pre-heated) until a final molar ratio of 145:1 DMPC:protein [4]. All reagents and instruments were pre-heated at 24 °C. Samples were gently mixed (for 5 s.) and clearance efficiency at 24 °C was determined as the time dependence of the light dispersion, monitoring absorbance (A) at 325 nm. Alternatively, spectrophotometers Helios Beta Single beam quartz coated (Thermo), or a UV–visible Agilent Cary 8454 (Agilent Technologies) was used yielding indistinguishable information.  All DMPC experiments were performed in the presence of 0.05% sodium azide. Each kinetics curve was normalized to the initial absorbance.  Curves were adjusted by fitting into a double exponential decay in the SigmaPlot software version 12.0. Efficiency of micellization (clearance) was determined as 1-A_325_ _nm_ at fitted curves at indicated times. Five different experiments were used to determine averaged 1-A_325_ _nm_ and standard deviation.

### 2.5 Lipoprotein particle construction

#### 2.5.1 By DMPC micellization

Proteins at 0.05 mg/mL were pre-incubated to a final temperature of 24 °C (as the rest of the elements: cuvettes, lipids, tubes, etc.), and, DMPC MLV were added at the same ratio as described above, gently mixed and incubated for either 3 or 72 h. This last condition was used to test the effect of temperature on particle storage. Complexes were incubated at room temperature (20 °C) or at −20 °C for 7 days. Samples were thawed at room temperature, and immediately seeded on gel for electrophoresis analysis.

#### 2.5.2 By detergent rearrangement

Lipoprotein particles were constructed using the rearrangement method mediated by detergents [1]. Sodium cholate in PBS was added to DMPC MLV to a final molar ratio of 40:60 (DMPC:sodium cholate). Once sodium cholate was added, initially “cloudy” DMPC MLV clarified by vortexing followed by 30-min incubation at 24 °C. Lipids were added to 0.05 mg/mL proteins to final molar ratio of 40 per each mol of protein, and samples were incubated overnight at 24 °C. Detergent was removed by exhaustive dialysis maintaining the mentioned temperature.

#### 2.5.3 Polyacrylamide gel electrophoresis

Home-made native gradient polyacrylamide gels (4–30%) were used to analyze lipoprotein particle size and relative amount. Each gel was imaged and transformed into an 8-bit image. If contrast or brightness was modified, it was applied to the entire gel. Densitometry and area of curves in plots were obtained by using ImageJ software version 1.51 j8. Data were normalized considering as 100% of density the sum of each lipoprotein band per lane. Triplicates were averaged and used to determine standard deviation for subsequent statistical analysis.

#### 2.5.4 Fast performance liquid chromatography (FPLC)

Lipoprotein particles were obtained for 72-h incubation of proteins at same DMPC:protein and protein concentration as described above. The relative size of the particles was estimated by size exclusion chromatography (SEC) using a Merck-Hitachi L6200 Intelligent pump. Samples were filtered with a 45 µm-pore syringe filter, eluted (300 µL each) through a Superose 6 HR 10/30 column, previously equilibrated with 50 mM Tris buffer pH 7.4 at a flow of 0.5 mL/min, and detected at 280 nm using a UV–VIS detector (Merck-Hitachi L4200). For better comparison curves registered as described above and normalized to maximum of each dataset. Only collected samples corresponding to chromatogram peaks were tested by PAGE.

#### 2.5.5 Transmission electron microscopy observations

Particle morphology (obtained after 72 h at 24 °C as described above) was characterized by transmission electron microscopy (TEM) on a JEOL1200 EX. Samples were seeded on Formvar grids, contrasted with 0.5% phosphotungstic acid and visualized by negative staining. Sample preparation for TEM imaging was performed at room temperature. Selected images were representative from seven independent images captured from different grid zones. Brightness and contrast were adjusted in ImageJ software version 1.51 j8 to improve the visualization of the lipoprotein's shape.

#### 2.5.6 Other analytical methods

For statistical analysis, datasets were analyzed in GraphPad Prism 8.0 software using parametric *t*-test with Welch´s correction or unpaired *T*-test corrected for multiple comparisons using the Holm-Sidak method. Only results with a significance level of *p*<0.05 were considered. Unless otherwise stated, measurements were reproduced in three independent experiments and reported as means of triplicates ± standard deviation.

## Ethics Statement

No animal or human samples have been used in this work.

## CRediT Author Statement

**Ivo Díaz Ludovico and Romina A. Gisonno:** Conceptualization, Methodology, Writing; Validation. **Horacio A. Garda, Marina C. Gonzalez**, Validation, Conceptualization. **Nahuel A. Ramella and M. Alejandra Tricerri**, Conceptualization, Writing, Funding acquisition, Methodology.

## Declaration of Competing Interest

Authors declare that they have not known competing financial interests or personal relationships which have, or could be perceived to have, influenced the work reported in this article
